# Editorial: Expanding knowledge on mineralized tissues in dental science: enamel, dentin, and pulp tissue

**DOI:** 10.3389/fdmed.2026.1814550

**Published:** 2026-03-11

**Authors:** Emi Shimizu, Maiko Suzuki, Motoki Okamoto, Hayato Ohshima

**Affiliations:** 1 Department of Oral Biology, Rutgers School of Dental Medicine, Newark, NJ, United States; 2 Department of Oral Science and Translational Research, College of Dental Medicine, Nova Southeastern University, Fort Lauderdale, FL, United States; 3 Department of Restorative Dentistry and Endodontology, Osaka University Graduate School of Dentistry, Osaka, Japan; 4 Division of Anatomy and Cell Biology of the Hard Tissue, Department of Tissue Regeneration and Reconstruction, Niigata University Graduate School of Medical and Dental Sciences, Niigata, Japan

**Keywords:** dental materials, genetics, mineralized tissue, regenerative medicine, tooth development

The mineralized tissues of the mouth—including enamel, dentin, pulp, and alveolar bone—play essential roles in dental health and have long been central to investigations in dental research. Recently, growing attention has been paid to the interactions between these hard tissues and their surrounding tissues, offering new perspectives on disease mechanisms and treatment strategies.

Regarding enamel, the classical concept of demineralization and remineralization mediated by oral bacteria, dietary substrates and saliva was established decades ago and continues to be an active focus of research. More recent advances include restorative approaches such as resin infiltration for managing partially demineralized enamel and biomimetic strategies designed to replicate the hierarchical structure of enamel.

In the research on the dentin-pulp complex, insights from bone biology have contributed to a better understanding of pulpal characteristics. At the same time, the development of dental materials with improved biocompatibility and sealing ability, particularly hydraulic calcium silicate–based materials, has renewed global interest in vital pulp therapy. This renewed interest stems from growing evidence that these materials can promote favorable biological responses, such as dentin bridge formation, pulpal healing, and reduced inflammatory activity. As a result, this progress has begun to challenge the traditional diagnostic distinction between reversible and irreversible pulpitis, suggesting that these terms may no longer fully reflect current biological and therapeutic concepts.

Accordingly, the present Research Topic, entitled “Expanding Knowledge on Mineralized Tissues in Dental Science: Enamel, Dentin, and Pulp Tissue,” aimed to integrate current knowledge on mineralized tissues involved in tissue formation and wound healing. This Research Topic comprises three original research articles and one systematic review and meta-analysis addressing tertiary dentin formation during pulpal wound healing, the filling quality of root canal sealers under different dentin moisture conditions, the effects of storage conditions on the enamel proteome, and the role of autologous peripheral venous blood concentrates in wound healing after tooth extraction.

Karaaslan et al. demonstrated that storage conditions substantially influence the enamel proteome, providing important implications for proteomic analyses of enamel. Because biological information is incorporated and preserved during enamel mineralization, extracted or exfoliated teeth can serve as records of health history and as resources for studying disease-related mechanisms. Their findings highlight that proteins such as serum albumin and enamel-specific proteins are sensitive to storage methods, emphasizing the importance of appropriate experimental design.

Yang et al. investigated root canal filling conditions in endodontic therapy, focusing on hydraulic calcium silicate–based sealers. They showed that canal drying during obturation largely depends on operator judgment and proposed clinically applicable procedures to standardize dentin moisture conditions using void as an objective criterion, addressing the lack of clear clinical guidance.

Nakano et al. evaluated the effects of n-butylboronic acid on immortalized human dental pulp cells and demonstrated positive regulation of cell proliferation and alkaline phosphatase activity. These findings support the growing interest in ion-mediated biological effects and suggest the potential for boron-releasing dental materials.

Meng et al. conducted a meta-analysis of 16 studies to assess the effectiveness of autologous peripheral venous blood concentrates in socket preservation. Their results showed more mature newly formed bone in the APVBC group than in spontaneous healing, with further enhancement when combined with graft materials, supporting their clinical utility in preventing alveolar bone resorption after tooth extraction.

Overall, this Research Topic provides integrated insights into mineralized tissues involved in tissue formation and wound healing. Calcification, as the final stage of differentiation and repair, remains incompletely understood across molecular, material, and clinical levels. By highlighting current knowledge gaps, we hope this Research Topic will guide future research and contribute to advances in both basic and clinical dental science.

